# Distinguishing Epileptiform Discharges From Normal Electroencephalograms Using Scale-Dependent Lyapunov Exponent

**DOI:** 10.3389/fbioe.2020.01006

**Published:** 2020-09-08

**Authors:** Qiong Li, Jianbo Gao, Qi Huang, Yuan Wu, Bo Xu

**Affiliations:** ^1^School of Computer, Electronics and Information, Guangxi University, Nanning, China; ^2^Center for Geodata and Analysis, Faculty of Geographical Science, Beijing Normal University, Beijing, China; ^3^Institute of Automation, Chinese Academy of Sciences, Beijing, China; ^4^International College, Guangxi University, Nanning, China; ^5^The First Affiliated Hospital of Guangxi Medical University, Nanning, China

**Keywords:** EEG, epileptiform discharges, power spectral density (PSD), scale-dependent Lyapunov exponent (SDLE), random forest classifier, support vector machine (SVM)

## Abstract

Epileptiform discharges are of fundamental importance in understanding the physiology of epilepsy. To aid in the clinical diagnosis, classification, prognosis, and treatment of epilepsy, it is important to develop automated computer programs to distinguish epileptiform discharges from normal electroencephalogram (EEG). This is a challenging task as clinically used scalp EEG often contains a lot of noise and motion artifacts. The challenge is even greater if one wishes to develop explainable rather than black-box based approaches. To take on this challenge, we propose to use a multiscale complexity measure, the scale-dependent Lyapunov exponent (SDLE). We analyzed 640 multi-channel EEG segments, each 4 *s* long. Among these segments, 540 are short epileptiform discharges, and 100 are from healthy controls. We found that features from SDLE were very effective in distinguishing epileptiform discharges from normal EEG. Using Random Forest Classifier (RF) and Support Vector Machines (SVM), the proposed approach with different features from SDLE robustly achieves an accuracy exceeding 99% in distinguishing epileptiform discharges from normal control ones. A single parameter, which is the ratio of the spectral energy of EEG signals and the SDLE and quantifies the regularity or predictability of the EEG signals, is introduced to better understand the high accuracy in the classification. It is found that this regularity is considerably greater for epileptiform discharges than for normal controls. Robustly having high accuracy in distinguishing epileptiform discharges from normal controls irrespective of which classification scheme being used, the proposed approach has the potential to be used widely in a clinical setting.

## 1. Introduction

Epilepsy is a common disorder of the brain (Li et al., 2019). Approximately 8–10% of people would experience an epileptic seizure during their lifetime (Gavvala and Schuele, 2016). In adults, the risk of the recurrence of seizure within the 5 years following a new-onset or a second seizure is 35 and 75%, respectively (Gavvala and Schuele, 2016). These percentages are even higher in children, with 50% of the recurrence within the 5 years following a single unprovoked seizure, and 80% after two unprovoked seizures (Camfield and Camfield, 2015). In the United States in 2011, about 1.6 million seizure patients made emergency department visits; approximately 25% of these visits were for new-onset seizures (Gavvala and Schuele, 2016). The exact incidence of epileptic seizures in low-income and middle-income countries is unknown, however it is speculated to exceed that in high-income countries (Ba-Diop et al., 2014).

Electroencephalography (EEG) provides a continuous measure of cortical function with excellent time resolution, and thus remains the primary diagnostic test of brain function, especially in those with epileptic seizures, even though new functional imaging procedures such as functional MRI (fMRI), single-photon emission computed tomography (SPECT), and positron emission tomography (PET) have been increasingly used for assessing anatomical changes in the brain. EEG is especially valuable in investigating patients with known or suspected seizures or encephalopathy. Seizures are however infrequent events in the majority of patients in an outpatient setting, making recording of ictal EEG time-consuming and labor intensive. So far, the mainstay of diagnosis remains to detect interictal (i.e., between seizures) epileptiform discharges. Therefore, epileptiform discharges are of fundamental importance in understanding the physiology of epilepsy. To aid in the clinical diagnosis, classification, prognosis, and treatment of epilepsy, it is critical to develop automated computer programs to distinguish epileptiform discharges from normal EEG.

Many methods have been developed to study EEG. Simple but important features of EEG include the amplitude values (Toet et al., 2005) and the Power Spectral Density (PSD) (Gao et al., 2007). Using wavelet transform is also a popular approach (Adeli et al., 2003; Subasi, 2007; Faust et al., 2015; Chen et al., 2017). Clinically, however, neurologists still rely heavily to visually examine the long continuous EEG signals. Unfortunately, this approach is time-consuming and prone to error due to human fatigue. This issue has motivated much effort to develop automated algorithms to detect epileptiform discharges or other features from EEG (Sharmila and Geethanjali, 2019). Among the notable works along this line are to use entropy (Nicolaou and Georgiou, 2012; Arunkumar et al., 2016, 2017) and complexity measures (Gao et al., 2011, 2012b; Martis et al., 2015; Medvedeva et al., 2016; Pratiher et al., 2016; Sikdar et al., 2018). The majority of the works published are however based on electrocorticogram (ECoG), which is invasively obtained by directly attaching electrodes to the cerebral cortex (Wang et al., 2019). Clinically, the more widely available form of EEG is the non-invasive scalp EEG. Compared with ECoG, scalp EEG signals are much poorer in terms of signal-to-noise ratios (Haufe et al., 2018). Scalp EEG recordings also contain various kinds of artifacts (Islam et al., 2016; Brienza et al., 2019), including eye movements (e.g., blinks), muscle activities (e.g., swallowing, head movements), and the heartbeat (Kappel et al., 2017). These noise and artifacts exacerbates greatly the difficulty in automatically detecting epileptiform discharges from normal controls. Although machine learning based approaches (Mirowski et al., 2008; Shen et al., 2009; Antoniades et al., 2016; Kuswanto et al., 2017; Ullah et al., 2018; van Putten et al., 2018; Subasi et al., 2019) can partly solve some of these problems, overall, the problem remains largely open, especially with regard to the development of explainable non-black-box based approaches.

In this paper, we propose to use scale-dependent Lyapunov exponent (SDLE) to develop a readily explainable approach to automatically detect epileptiform discharges from normal controls. SDLE is a multiscale complexity measure developed to unambiguously distinguish chaos from noise, and more fundamentally to automatically characterize the defining parameters/properties of complex data (Gao et al., 2006, 2007). SDLE stems from two important concepts, the time-dependent exponent curves (Gao and Zheng, 1993, 1994a,b; Gao, 1997) and the finite size Lyapunov exponent (Torcini et al., 1995; Aurell et al., 1996, 1997). SDLE was first introduced in Gao et al. (2006, 2007), and has been further developed in Gao et al. (2009, 2012a) and applied to characterize ECoG (Gao et al., 2011), HRV (Hu et al., 2009, 2010), financial time series (Gao et al., 2013), Earth's geodynamo (Ryan and Sarson, 2008), precipitation dynamics (Fan et al., 2013), sea clutter (Hu and Gao, 2013), THz imagery (Blasch et al., 2012), and randomness (Li et al., 2016). We will show that the proposed approach is very accurate in distinguishing epileptiform discharges from normal controls.

The remainder of the paper is organized as follows. In section 2, we briefly describe the EEG data and analysis methods. In section 3, we present analysis results. In section 4, we summarize our findings.

## 2. Materials and Methods

### 2.1. Data

The scalp EEG data analyzed here were clinically obtained at the First Affiliated Hospital to Guangxi Medical University. The studies involving human participants were reviewed and approved by the ethics committee of the First Affiliated Hospital to Guangxi Medical University. The participants provided their written informed consent to participate in this study. Fifty-nine epilepsy patients underwent a 3-h video-EEG monitoring with 19-channel EEG recording with electrodes placed on the scalp under the international 10–20 system at 256 Hz sampling rate. The electrode impedances were kept below 10*K*Ω. The 19 scalp electroencephalographic electrodes were arranged according to the names *Fp*1, *Fp*2, *F*7, *F*3, *Fz*, *F*4, *F*8, *T*3, *C*3, *Cz*, *C*4, *T*4, *T*5, *P*3, *Pz*, *P*4, *T*6, *O*1, and *O*2. Since the information yielded by an EEG channel is essentially the difference of electrical activity between two electrodes in the time-domain (Pardey et al., 1996; Lopez et al., 2016), the amplitude, frequency, and synchronization of the brain waves and background will change (Seeck et al., 2017; Vanherpe and Schrooten, 2017), depending on which montage is chosen (e.g., earlobe reference, averaged reference, or bipolar; Christodoulakis et al., 2013; Geier and Lehnertz, 2017; Rana et al., 2017; Acharya and Acharya, 2019; Rios et al., 2019). In this work, we choose the widely used earlobe reference.

**Figure 1:**
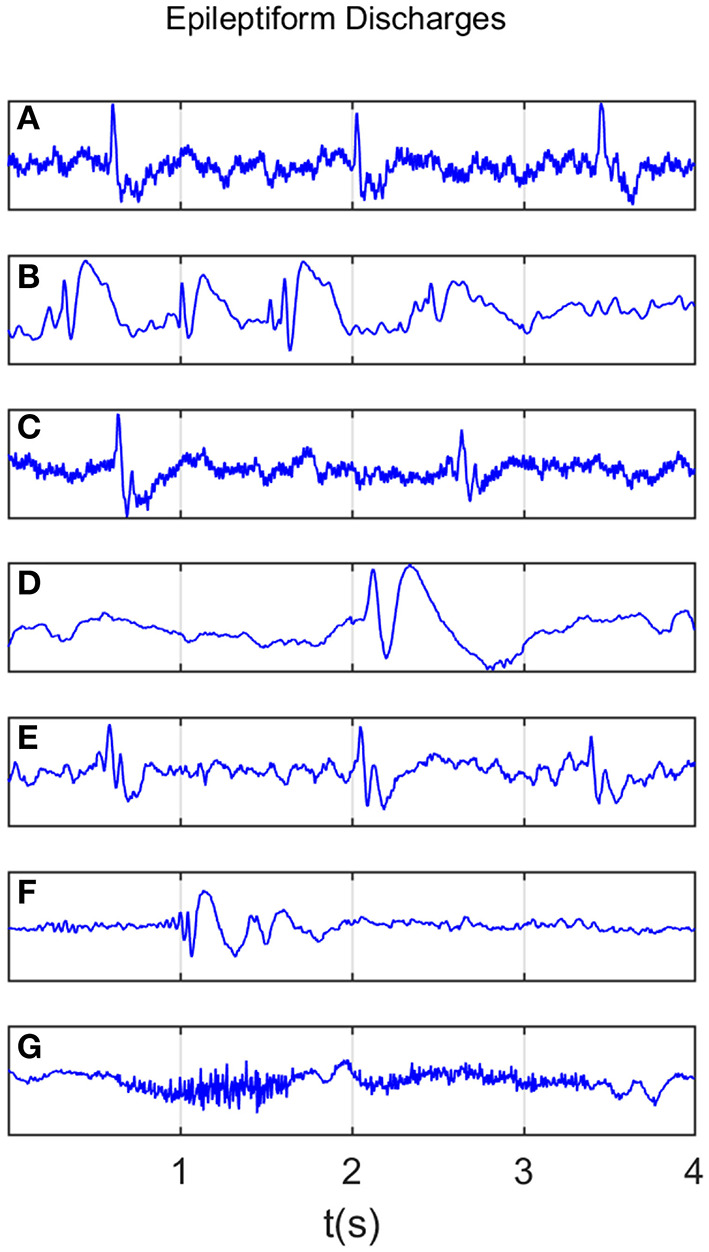
Typical waveforms of the 7 major epileptiform EEG, where **(A-G)**, denotes spike wave, spike and slow wave complex, sharp wave, sharp and slow wave complex, polyspike complex, polyspike and slow wave complex, spike rhythm discharges, respectively.

All epileptiform discharges were annotated by an experienced clinical neurophysiologist based on the average montage with an analog bandwidth of 0.1~70 *Hz* and a notch filter of 50 *Hz*. EEG signals were segmented into 4 *s* epochs, with each epoch assigned a random number. The collected epochs were transformed into European Data Format (EDF) for further analysis. In total, there were 532 EEG recordings of epileptiform discharges and 100 healthy controls, each 4 *s* long, from all the participants. Among the 532 short epileptic discharges, there were 69 spike waves, 82 sharp waves, 174 spike and slow wave complexes, 72 sharp and slow wave complexes, 64 polyspike complexes, 77 polyspike, and slow wave complexes and 2 spike rhythmic discharges. Note the numbers for these seven epileptiform discharges sum up to 540, which is slightly larger than 532. The reason is a few discharges were considered to simultaneously belong to more than 1 of the 7 different epileptiform discharges. For convenience of referencing, the definitions for these 7 epileptiform discharges are given below, together with the number of cases analyzed for each type indicated in the parentheses immediately following each terminology. Examples of their waveforms are shown in Figure 1.

- Spike wave (69): the most basic paroxysmal EEG activity, with a duration of 20~70 *ms*; amplitude varies but typically >50 *uV* (Kane et al., 2017).
- Sharp wave (82): a transient wave similar to the spike and clearly distinguishable from background activity; its time limit is 70~200 *ms* (5~14 *Hz*), amplitude is between 100 and 200 *uV*, and the phase is usually negative.
- Spike and slow wave complex (174): pattern consisting of a spike followed by a slow wave (classically the slow wave being of higher amplitude than the spike); may be single or multiple (Kane et al., 2017).
- Sharp and slow wave complex (72): pattern consisting of a sharp followed by a slow wave (classically the slow wave being of higher amplitude than the sharp); may be single or multiple (Kane et al., 2017).
- Polyspike complex (64): a sequence of two or more spikes.
- Polyspike and slow wave complex (77): pattern with two or more spikes associated with one or more slow waves.
- Spike rhythm (2): a rare pattern of widespread 10~25 *Hz* spike rhythm outbreak, with an amplitude of 100~200 *uV* and the highest voltage in the frontal area, lasting more than 1 *s*.

Recall that a few epileptiform discharge waveforms were considered to simultaneously belong to more than 1 of these 7 different epileptiform discharges. Because of this, we will not pursue the issue of further characterizing the differences among the 7 epileptiform discharges here.

### 2.2. Computation of Power Spectral Density (PSD)

PSD of EEG can be readily obtained by taking Fourier transform of the EEG signal, computing the square of the amplitude of the transform to obtain the power, and finally plotting the power against the frequency. In clinical applications, brain waves are often categorized into five bands: delta (0.5~ 3*Hz*), theta (4~7 *Hz*), alpha (8~13 *Hz*), beta (14~30 *Hz*), and gamma (>30 *Hz*), respectively. To obtain the energy of these waves, one only needs to integrate the PSD curve over the respective wave band. In this work, we integrate the PSD curve for frequencies between 0.5 and 25 Hz for the 10 electrodes with the strongest signals, and then take the average.

### 2.3. Computation of the SDLE

As with the estimation of PSD, for each subject, we picked up 10 strongest EEG signals from 19 electrodes, computed SDLE from each one of the 10 EEG signals, and took the average.

**Figure 2:**
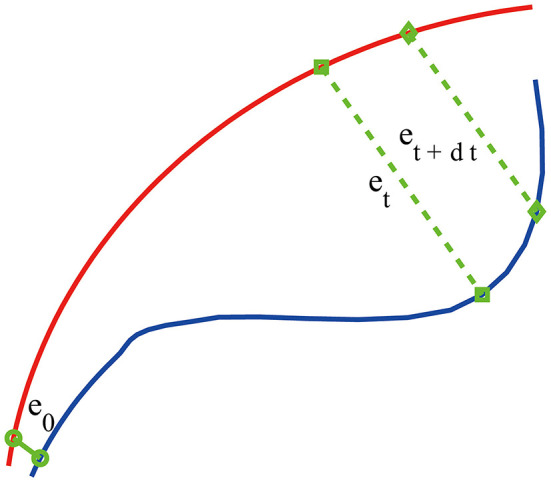
A schematic showing two arbitrary trajectories in a general high-dimensional space, with the distance between them at time 0, *t*, and *t*+δ*t* being ϵ_0_, ϵ_*t*_, and ϵ_*t*+δ*t*_, respectively.

To compute SDLE, we first need to reconstruct a phase space from the EEG signals. Denote the signal as *x*(*i*), *i* = 1, ⋯ , *n*, we construct vectors

(1) $$\begin{array}{r} V_{i}=\left[x\left(i\right),x\left(i+L\right),...,x\left(i+\left(m-1\right)L\right)\right], \end{array}$$

where *m* is called the embedding dimension and *L* the delay time. In practice, *m* and *L* have to be chosen properly. This is the issue of optimal embedding. For example, to reconstruct the phase plane of a harmonic oscillator from a sinusoidal signal, the optimal delay time is 1/4 of the period (Gao et al., 2007). Extensive works have been done to optimally determine *m* and *L*. Two of the most systematic and most extensively tested approaches are a statistical method called the false nearest neighbor method (Kennel et al., 1992) and a dynamical method based on time-dependent exponent curves developed by Gao and Zheng (1993, 1994a,b). The basic idea of the latter is to choose *L* in such a way that the motion in the reconstructed phase space is as uniform as possible (in the case of a harmonic oscillator, the reconstructed phase plane is an ellipse, which becomes a circle when *L* is 1/4 of the period; motion on the circle is the most uniform when compared with motions on ellipses). This is achieved by requiring divergence characterized by time-dependent exponent curves be a minimum when *L* is varied, and the divergence does not become much larger when *m* is further increased. This is the method that is employed here. For the EEG signals analyzed in this work, which was sampled with a sampling frequency of 256 Hz, we found *L* = 1 is optimal. With larger sampling frequency, *L* also has to be larger. For example, when the sampling frequency is 1,024 Hz, *L* then needs to be 4. As our EEG signal is not that long (4 *s*, or 1,024 points), we also found that *m* = 2 worked very well. After the phase space is reconstructed, we consider an ensemble of trajectories. We denote the initial separation between two nearby trajectories by ϵ_0_, and their *average separation* at time *t* and *t*+Δ*t* by ϵ_*t*_ and ϵ_*t*+Δ*t*_, respectively. The trajectory separation is schematically shown in Figure 2. Note ϵ_*t*+Δ*t*_ is not necessarily larger than ϵ_*t*_. We then examine the relation between ϵ_*t*_ and ϵ_*t*+Δ*t*_, where Δ*t* is small. When Δ*t* → 0, we have,

(2) $$\begin{array}{r} \epsilon_{t+\Delta t}=\epsilon_{t}e^{\lambda \left(\epsilon_{t}\right)\Delta t}, \end{array}$$

where λ(ϵ_*t*_) is the SDLE given by

(3) $$\begin{array}{r} \lambda \left(\epsilon_{t}\right)=\frac{\ln\;\epsilon_{t+\Delta t}-\ln\;\epsilon_{t}}{\Delta t}. \end{array}$$

With the above definition, we can readily compute SDLE using the vectors defined by Equation (1). Specifically, we check whether pairs of vectors (*V*_*i*_, *V*_*j*_) satisfy the following Inequality:

(4) $$\begin{array}{r} \epsilon_{i}\leq ||V_{i}-V_{j}||\leq \epsilon_{i}+\Delta \epsilon_{i},\;i=1,2,3,\cdots \;, \end{array}$$

where ϵ_*i*_ and Δϵ_*i*_ are prescribed small distances. Geometrically, a pair of ϵ_*i*_ and Δϵ_*i*_ defines a shell, with the former being the diameter of the shell and the latter the thickness of the shell (which reduces to a ball with radius Δϵ_*k*_ when ϵ_*k*_ = 0; in a 2-D plane employed here, a ball is a circle described by ${\left(x_{i}-a\right)}^{2}+{\left(x_{i+1}-b\right)}^{2}=r^{2}$, where (*a, b*) is the center of the circle, and *r* is the radius). We then monitor the evolution of all such vector pairs (*V*_*i*_, *V*_*j*_) within a shell and take the ensemble average over the indices *i, j*. Since we are most interested in exponential or power-law functions, we assume that taking logarithm and averaging can be exchanged, then Equation (3) can be written as

(5) $$\begin{array}{l} \lambda (\epsilon_{t})=\frac{\ln\langle \left\|V_{i+t+\Delta t}-V_{j+t+\Delta t}\right\|\rangle -\ln\langle \left\|V_{i+t}-V_{j+t}\right\|\rangle }{\Delta t}\\ \;\approx \frac{\langle \ln\|V_{i+t+\Delta t}-V_{j+t+\Delta t}\|-\ln\|V_{i+t}-V_{j+t}\|\rangle }{\Delta t} \end{array}$$

where *t* and Δ*t* are integers in units of the sampling time, the angle brackets denote the average over indices *i, j* within a shell. Note 〈||*V*_*i*+*t*+Δ*t*_−*V*_*j*+*t*+Δ*t*_||〉 and 〈||*V*_*i*+*t*_−*V*_*j*+*t*_||〉 amount to ϵ_*t*+Δ*t*_ and ϵ_*t*_, respectively. For EEG signals, the most relevant scaling law for SDLE is

(6) $$\begin{array}{r} \lambda \left(\epsilon \right)\sim -\gamma \ln\;\epsilon , \end{array}$$

where γ determines the speed of loss of information.

To make the computation of SDLE readily repeated by other researchers, and more importantly, to enable different researchers to readily compare their results, we recommend to use the size of the first shell by $1/\sqrt{10}$ of the standard deviation of the EEG signal, and successive shells shrink by a factor of $1/\sqrt{2}$. Altogether, we used four shells, and then took the average of the four SDLE curves.

### 2.4. Random Forest Classifier (RF)

Random forest (RF) is a learning technique for classification based on ensembles (Cutler et al., 2012). It is not affected by overtraining, does not require normalization of the input data, and has high accuracy. It uses many separate classification trees. Each tree is obtained through a separate bootstrap sample from the data set and classifies the data. A majority vote among the trees provides the final result.

The objective of the RF classifier used here is to classify which of the two classes an EEG signal belongs to: normal or epileptiform discharges. The inputs to the RF classifier are the PSD and a feature extracted from the SDLE curve. Following usual practice, we have randomly taken one-third of the total data as testing data and two-thirds of the data for training the model in this paper.

### 2.5. Support Vector Machine (SVM)

Support Vector Machine (SVM) is a popular machine learning method for pattern classification (Cristianini and Shawe-Taylor, 2000). It has been widely used in biomedical applications. It aims to find a hyperplane in an *N*-dimensional space (*N*, the number of features) that maximizes the distance between two classes of points. Hyperplanes are decision boundaries that help classify the data points. Data points falling on either side of the hyperplane can be attributed to the two different classes. The dimension of the hyperplane depends upon the number of features. If the number of input features is 2, then the hyperplane is just a line. If the number of input features is 3, then the hyperplane is a two-dimensional plane. When the number of features exceeds 3, it becomes difficult to imagine the shape of the hyperplane, nevertheless, it can be readily computed.

### 2.6. Evaluation of Performance

The consistency between the diagnosis by the neurologists and machine classification needs to be quantified. This can be accomplished by computing the receiver operating characteristic (ROC) curve and many statistics derived from the ROC curve. A good understanding of these metrics can be based on the confusion matrix, which is a table with two rows and two columns that reports the number of false positives (FP), false negatives (FN), true positives (TP), and true negatives (TN). From them we can define three major metrics:

(7) $$\begin{array}{r} sensitivity=\frac{TP}{TP+FN} \end{array}$$

(8) $$\begin{array}{r} specificity=\frac{TN}{TN+FP} \end{array}$$

(9) $$\begin{array}{r} accuracy=\frac{TP+TN}{TP+FP+TN+FN} \end{array}$$

Note that the sensitivity is also called true positive rate (TPR) and 1−*specificity* is also called false positive rate (FPR).

The ROC is a plot of TPR vs. FPR using different threshold values as a sweeping variable. The ROC is a good way to characterize imbalanced data sets, as it does not suffer from class imbalance. The area below the ROC is called area under curve (AUC). Its value takes from 0 to 1. A value of AUC being 0.5 means the classification model has no predictive ability at all. On the other hand, when the value of AUC reaches 1, the prediction ability is 100%. This is equivalent to the ROC being a unit step function.

## 3. Result

**Figure 3:**
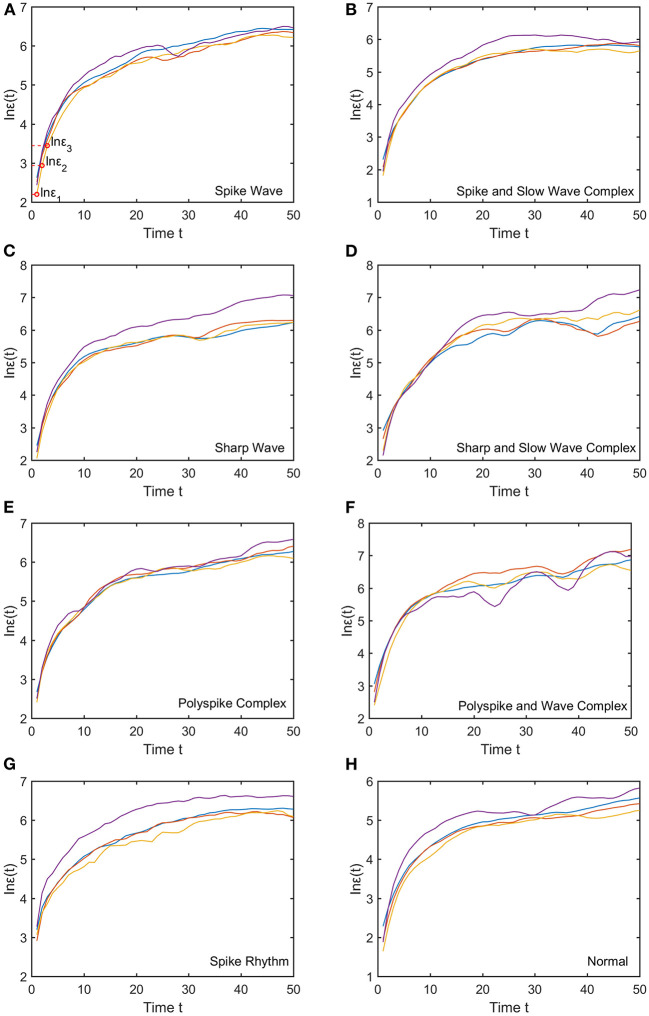
Typical ln ε_*t*_ vs. *t* curves for epileptiform discharges and normal EEG, where the four curves correspond to four different shells, with the diameter of the largest shell being $1/\sqrt{10}$ of the standard deviation of the EEG signal, and successive shells shrinking by a factor of $1/\sqrt{2}$. **(A–H)** illustrates the different between the seven types of epileptiform discharges (spike wave, spike and slow wave complex, sharp wave, sharp and slow wave complex, polyspike complex, polyspike and slow wave complex, spike rhythm discharges) and normal EEG.

**Figure 4:**
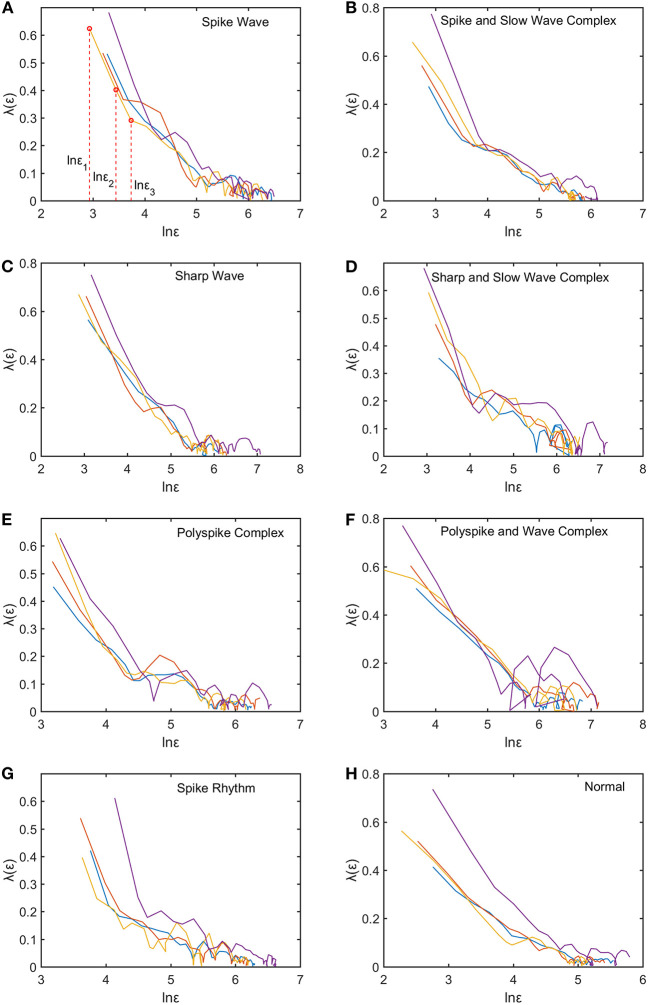
Typical λ(ϵ) vs. lnϵ curves for epileptiform discharges and normal EEG. The four curves represented in four different colors correspond to the error growth curves shown in Figure 3. **(A–H)** illustrates the different between the seven types of epileptiform discharges (spike wave, spike and slow wave complex, sharp wave, sharp and slow wave complex, polyspike complex, polyspike and slow wave complex, spike rhythm discharges) and normal EEG.

We mentioned that for each subject, to compute the SDLE curves, we chose from the 19 electrodes 10 strongest EEG signals, computed the SDLE curves from each EEG signal, then took the average. For each EEG signal, we reconstructed a phase space with *m* = 2, *L* = 1, then computed 4 ln ϵ_*t*_ vs. *t* curves corresponding to 4 shells, with the diameter of the largest shell being $1/\sqrt{10}$ of the standard deviation of the EEG signal, and successive shells shrinking by a factor of $1/\sqrt{2}$. Eight typical ln ε_*t*_ vs. *t* curves for epileptiform discharges and normal EEG corresponding to these four shells were shown in Figure 3. For simplicity, we call these error growth curves. Note the classic algorithm of computing the Lyapunov exponent amounts to assuming $\epsilon_{t}\sim \epsilon_{0}e^{\lambda_{1}t}$, where λ_1_ is the largest positive Lyapunov exponent, and estimating λ_1_ by (ln ϵ_*t*_−ln ϵ_0_)/*t* (Wolf et al., 1985). This clearly is inappropriate here since ln ϵ_*t*_ does not increase with *t* linearly. In other words, small variations in EEG signals did not really grow exponentially. This difficulty is readily overcome with SDLE, since the latter is the local slopes of such error growth curves, which are always well-defined. The SDLE curves corresponding to the error growth curves of Figure 3 were shown in Figure 4. There are 4 SDLE curves here, corresponding to 4 shells chosen. The left-most curve corresponds to the smallest shell, while the right-most curve corresponds to the largest shell (they often are indistinguishable on larger scales). The most salient feature of these SDLE curves is the scaling behavior described by Equation (6).

It would be desirable to combine the 4 SDLE curves into a single curve. The most rigorous way to estimate the SDLE at a specific scale ϵ* is to first interpolate each SDLE curve to that scale so that it has a value there, then average the 4 SDLE curves at ϵ* using the number of pairs of vectors in each shell as the weights. For simplicity, one could also first align the 4 SDLE curves with the left-most curve, and then simply take the arithmetic average (in cases where the 4 curves are indistinguishable, then this alignment operation is unnecessary). To make the proposed method easier to reproduce, we adopted this simplified approach here. For the purpose of distinguishing epileptiform discharges from normal controls, we focused on three SDLEs λ(ϵ_1_), λ(ϵ_2_), and λ(ϵ_3_) at three specific scales ϵ_1_, ϵ_2_, and ϵ_3_, and their average, which was denoted as $\bar{\lambda }\left(\epsilon \right)$. The three scales ϵ_1_, ϵ_2_, and ϵ_3_ were specifically indicated in Figures 3A, 4A. These scales correspond to the smallest, intermediate, and boundary scales where the scaling law of Equation (6) holds.

**Figure 5:**
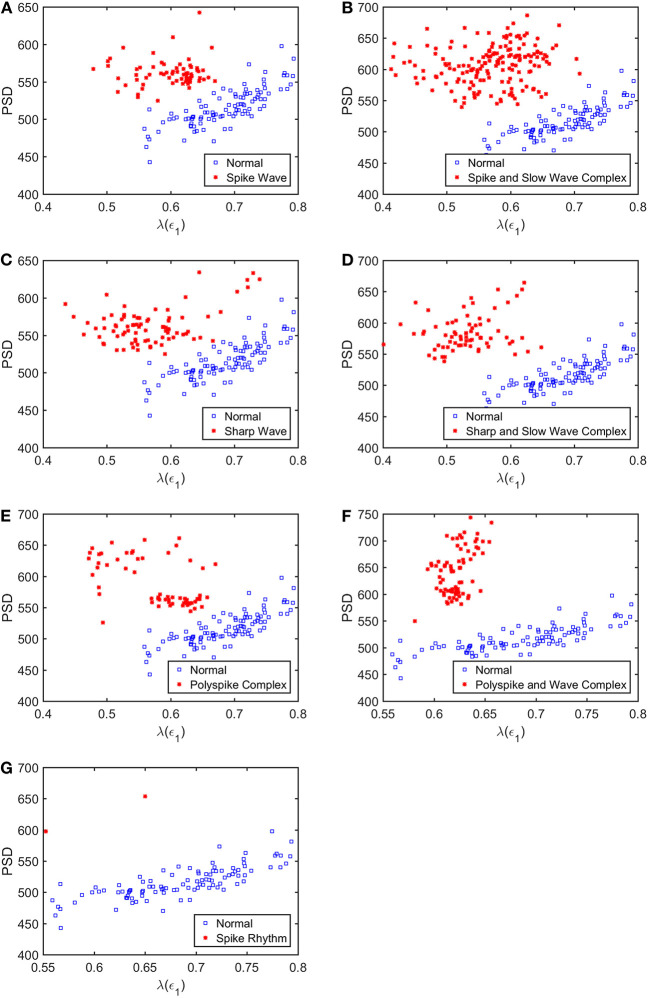
Scatter plots with PSD and λ(e1), where **(A–G)**, illustrates the different between the seven types of epileptiform discharges (spike wave, spike and slow wave complex, sharp wave, sharp and slow wave complex, polyspike complex, polyspike and slow wave complex, spike rhythm discharges) and normal EEG. These plots highly suggest the classification accuracy will be very high.

**Figure 6:**
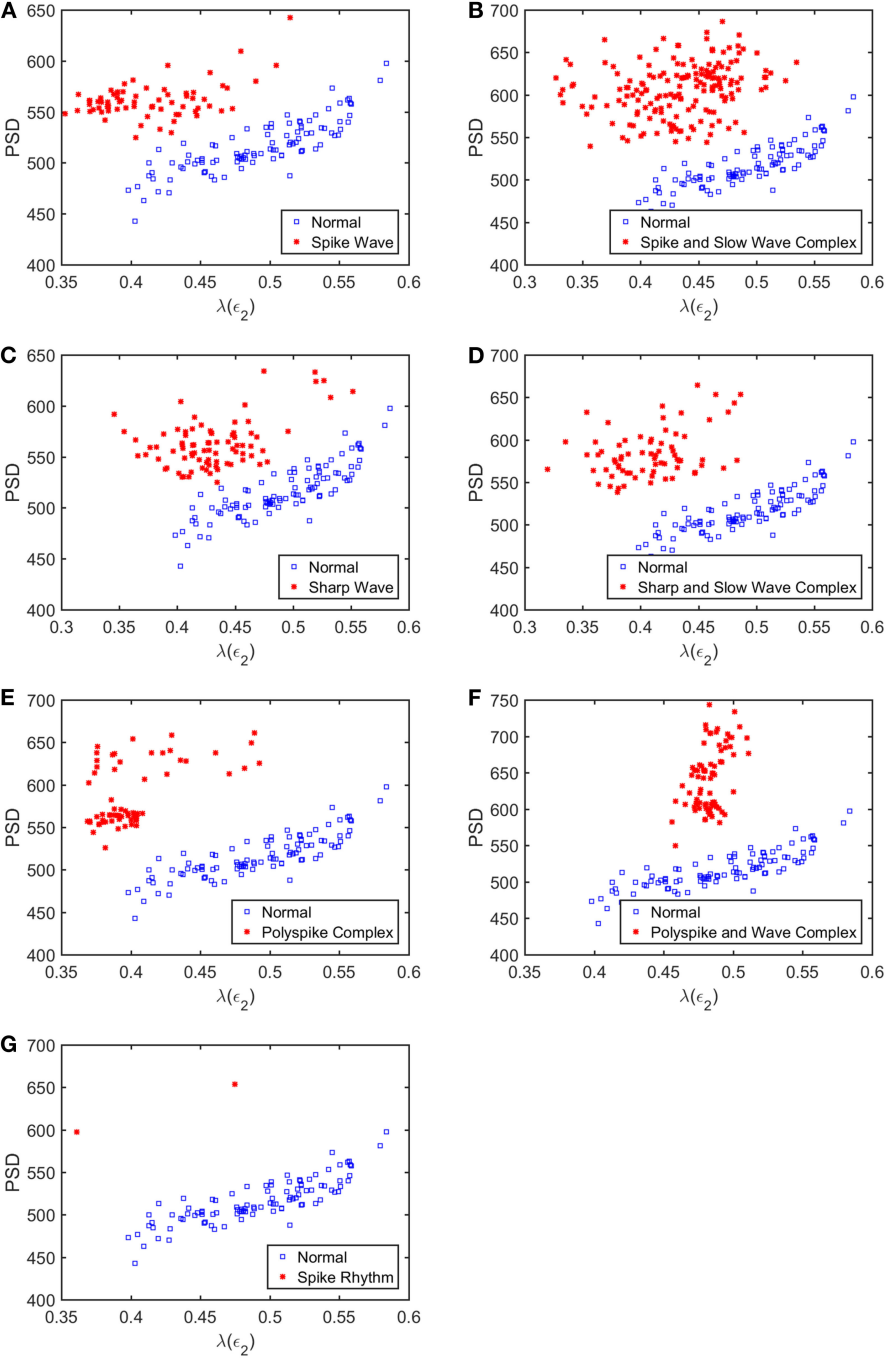
Scatter plots with PSD and λ(e2), where **(A–G)**, illustrates the different between the seven types of epileptiform discharges (spike wave, spike and slow wave complex, sharp wave, sharp and slow wave complex, polyspike complex, polyspike and slow wave complex, spike rhythm discharges) and normal EEG. These plots highly suggest the classification accuracy will be very high.

**Figure 7:**
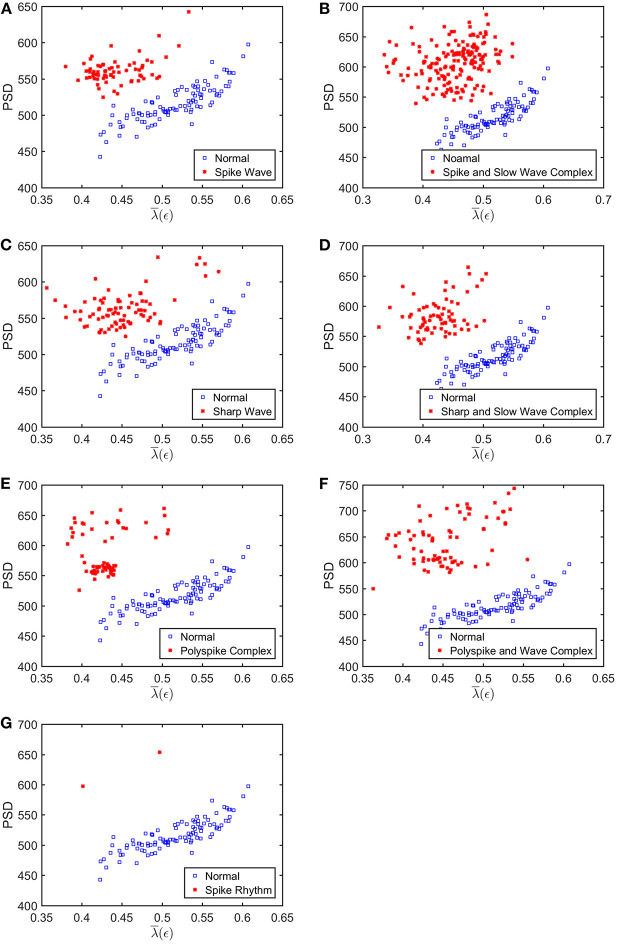
Scatter plots with PSD and $\bar{\lambda }\left(\epsilon \right)$, where **(A–G)**, illustrates the different between the seven types of epileptiform discharges (spike wave, spike and slow wave complex, sharp wave, sharp and slow wave complex, polyspike complex, polyspike and slow wave complex, spike rhythm discharges) and normal EEG. These plots highly suggest the classification accuracy will be very high.

To appreciate how well SDLEs can be used to distinguish epileptiform discharges from normal controls, we formed scatter plots with PSD and SDLEs, where PSD was obtained using Fourier transform, as we explained earlier. The scatter plots with PSD and λ(ϵ_1_), PSD and λ(ϵ_2_), and PSD and $\bar{\lambda }\left(\epsilon \right)$ were shown in Figures 5–7, respectively. We observe that in all these three cases, the separation between all seven types of epileptiform discharges and the normal control was excellent. Therefore, we can expect that the classification accuracy will be very high. Below, we specifically evaluate the performance of these three algorithms, which use PSD and λ(ϵ_1_), PSD and λ(ϵ_2_), and PSD and $\bar{\lambda }\left(\epsilon \right)$, respectively.

To compute the classification accuracy, we employed RF and SVM. We randomly took two-thirds of the data as the training data and the remaining one-third of the total data as the testing data. The class distribution of the samples in the training and testing data set is summarized in Table 1. The test performance of the classifier can be determined by computing the metrics defined in section 2.6. The confusion matrix in Table 2 for Algorithm 1, which used PSD and λ(ϵ_1_), showed that 1 out of 34 normal subjects was classified incorrectly by the two classification algorithms RF and SVM as the epileptiform discharge, and 1 out of 180 epileptiform discharges was classified incorrectly as the normal subject by RF and SVM. Algorithm 2, which used PSD and λ(ϵ_2_), was even better, which only misclassified 1 out of 180 epileptiform discharges as a normal subject by the RF, but without any other errors (the classification accuracy remained the same as that for Algorithm 1 when SVM is used). Algorithm 3, which used PSD and $\bar{\lambda }\left(\epsilon \right)$, was also excellent, which only misclassified 1 out of 34 normal subjects as an epileptiform discharge, but without any other errors for both RF and SVM. These were also summarized in Table 2. With these confusion matrices, we computed Sensitivity, Specificity, and Accuracy of these three algorithms. They were listed in Table 3. We find that all the three algorithms are excellent, with their accuracy all exceeding 99%, for both classification schemes RF and SVM.

**Table 1:** Class distribution of the samples in the training and testing data sets.

**Classes**	**Training set**	**Testing set**	**Total**
Normal controls	66	34	100
Epileptiform discharges	360	180	540
Total	426	214	640

**Table 2:** Confusion Matrix for the testing data of 180 epileptiform discharges and 34 normal controls: Algorithms 1, 2, 3 use PSD and λ(ϵ_1_), PSD and λ(ϵ_2_), PSD and $\bar{\lambda }\left(\epsilon \right)$, respectively.

**Classifier**	**Algorithms**	**Result**	**Epileptiform discharges**	**Healthy controls**
RF	Algorithm 1	Epileptiform discharges	179	1
		Healthy controls	1	33
	Algorithm 2	Epileptiform discharges	179	1
		Healthy controls	0	34
	Algorithm 3	Epileptiform discharges	180	0
		Healthy controls	1	33
SVM	Algorithm 1	Epileptiform discharges	179	1
		Healthy controls	1	33
	Algorithm 2	Epileptiform discharges	179	1
		Healthy controls	1	33
	Algorithm 3	Epileptiform discharges	180	0
		Healthy controls	1	33

**Table 3:** Classification performance measures.

**Classifier**	**Algorithms**	**Sensitivity (%)**	**Specificity (%)**	**Accuracy (%)**	**AUC**
RF	Algorithm 1	99.44	97.06	99.07	0.9784
	Algorithm 2	99.44	100.00	99.53	0.9980
	Algorithm 3	100.00	97.06	99.53	0.9953
SVM	Algorithm 1	99.44	97.06	99.07	0.9766
	Algorithm 2	99.44	97.06	99.07	0.9727
	Algorithm 3	100.00	97.06	99.53	0.9953

**Figure 8:**
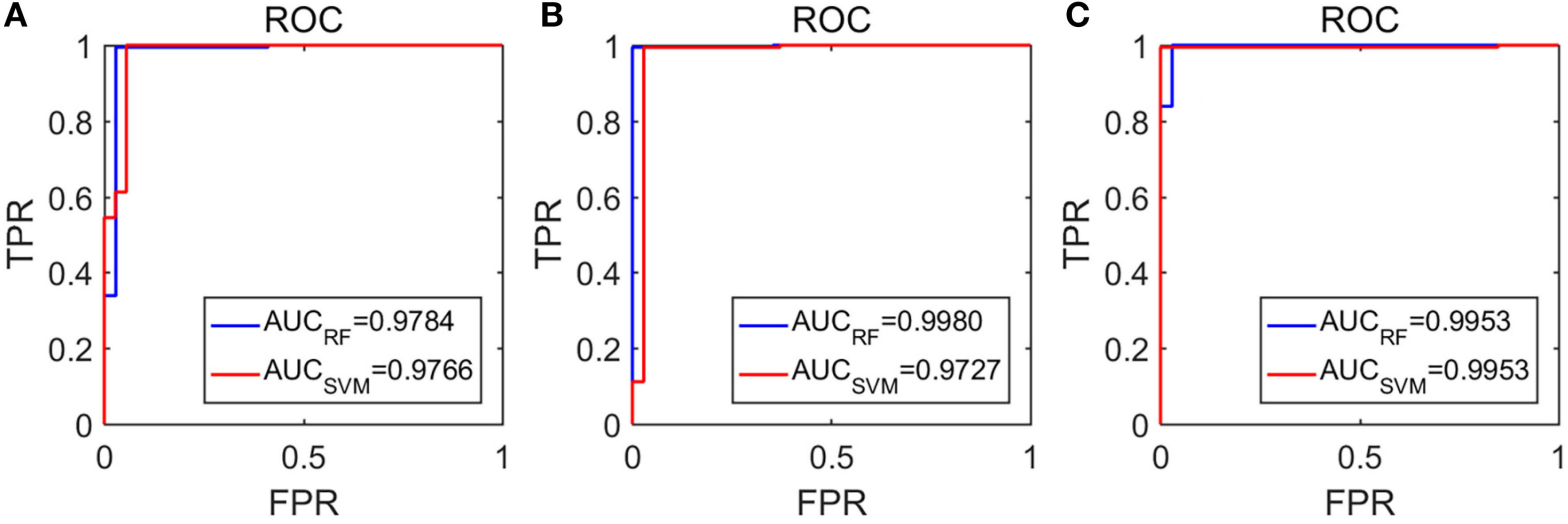
The ROC curves for the testing data: **(A–C)** are for algorithms using PSD and λ(ϵ_1_), PSD and λ(ϵ_2_), and PSD and $\bar{\lambda }\left(\epsilon \right)$, respectively.

The amazing performance of these methods can be further corroborated by the unit step function like ROC curves shown in Figure 8. To facilitate comparison of our algorithms with that of Anh-Dao et al. (2018), which achieved a high AUC of 0.945, we also listed the AUC for the three algorithms proposed here in Table 3. The AUC of the three algorithms proposed here ranges from 0.9727 to 0.9980, and therefore, are all considerably better than that of Anh-Dao et al. (2018).

## 4. Conclusion and Discussion

In this paper, we have proposed to employ SDLE for distinguishing epileptiform discharges from normal EEGs, with the aim of being able to use them conveniently in a clinical setting. We found that SDLE computed from scalp EEG signals was mainly characterized by a scaling law described by Equation (6). When the scale parameters were confined to where this scaling law held, SDLE was very effective in distinguishing epileptiform discharges from normal EEG. Using RF and SVM, the proposed approach with different features from SDLE was found to robustly achieve an accuracy exceeding 99% in distinguishing epileptiform discharges from normal control ones.

What is the reason that the choice of concrete classification schemes such as RF or SVM is not critical for the proposed approach to have high accuracy in distinguishing epileptiform discharges from normal control ones? It has to be because of the excellent separations revealed by the scatter plots shown in Figures 5–7. To better understand the explainability of the proposed approach, we need to understand better the meaning of the SDLE. The definition of SDLE is equivalent to

(10) $$\begin{array}{r} \ln\;\epsilon_{t}=\ln\;\epsilon_{0}+\int_{0}^{t}\lambda \left(\epsilon_{t}\right)dt. \end{array}$$

Letting ϵ_*T*_*db*__ = 2ϵ_0_, we find the error doubling time *T*_*db*_ given by

(11) $$\begin{array}{r} \ln\;2=\int_{0}^{T_{db}}\lambda \left(\epsilon_{t}\right)dt. \end{array}$$

As the first approximation, we may consider 1/λ(ϵ) to be proportional to the error doubling time (Gao et al., 2009). This understanding motivates us to combine the two parameters PSD and SDLE into a single parameter such as PSD/λ(ϵ_1_). Since on average PSD is larger but λ(ϵ_1_) (as well as λ(ϵ_2_) and $\bar{\lambda }\left(\epsilon \right)$, as shown in Figures 5–7 is smaller for epileptiform discharges than for normal control ones,

we can expect that this ratio will be on average larger for epileptiform discharges. In fact, this ratio can be regarded as a measure of the regularity or predictability of EEG signals, since large PSD stems from synchronized firing of neurons, while small SDLE highlights slow divergence and thus considerable regularity and predictability.

**Figure 9:**
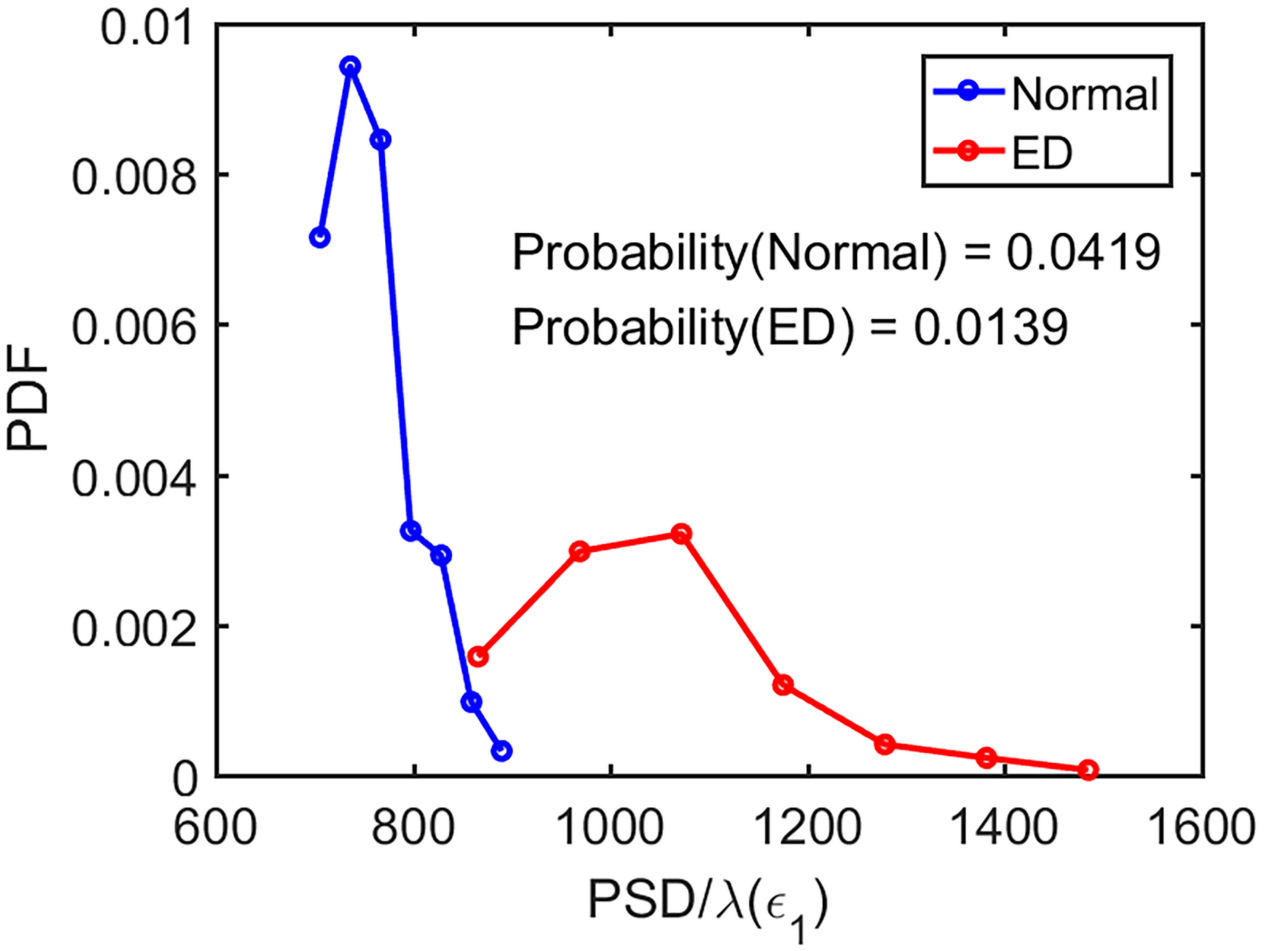
The probability density distribution (PDF) for the ratio PSD/λ(ϵ_1_) of the epileptiform discharges (red curve) and normal control ones (blue curve). The overlapping of the blue and the red curves defines a right and left tail for the blue and red curves, respectively; the corresponding probabilities for them are 1.39 and 4.19%, as indicated in the plot.

Now the question is whether such a single parameter can effectively distinguish normal control ones from epileptiform discharges. For this purpose, we have computed the probability density distribution (PDF) for PSD/λ(ϵ_1_) of the epileptiform discharges and the normal control ones. The results are shown in Figure 9 as the blue and the red curves, respectively. The overlapping of the blue and the red curves defines a right and a left tail for the blue and the red curves; the corresponding probabilities for them are 1.39 and 4.19%, as indicated in the plot. They correspond to the probability that a normal control one may be misclassified as an epileptiform discharge and vice versa. As the classification accuracy with the scheme based on a single parameter will not be higher than that based on two parameters, we can readily understand that the probabilities of 1.39 and 4.19% are the lower bounds that a normal control may be misclassified as epileptiform discharges, and vice verse. This is surely consistent with the probabilities shown in Table 3 (the case for Algorithm 1). As these misclassification probabilities are very low, we thus can be confident that the proposed approach will always be very accurate in distinguishing epileptiform discharges from normal control ones, no matter what classification schemes are used for classification.

It is interesting to note that if we choose SDLE corresponding to larger scales, such as ϵ_3_ indicated in Figures 3A, 4A, an algorithm based on PSD and λ(ϵ_3_) would be slightly worse than the three algorithms discussed here, but still slightly better than that of Anh-Dao et al. (2018). This suggests the importance of properly selecting the scale for analysis. On the other end, if we use a three parameter method, for example, using PSD, $\bar{\lambda }\left(\epsilon \right)$, and ϵ_∞_ (which characterizes the size of an attractor and amounts to the largest scale in Figure 3), then the accuracy in distinguishing epileptiform discharges from normal controls can be further improved to 100%. The reason is that ϵ_∞_ contains information independent of PSD and SDLE. However, we had not further pursed the issue of improving the accuracy here, since the high accuracy achieved by the easily explainable algorithms presented is already more than satisfying. Overall, our analysis highly suggests that the proposed approach is very promising to be used clinically.

It is worth noting that the epileptiform discharges analyzed here were provided by our collaborators at Guangxi Medical University in two batches: in the first batch, which was about 2/3 of the data analyzed here, the accuracy was similar to that reported here. Then another 1/3 of the data were given to us to further examine whether the accuracy remained as high. It was yes. Nevertheless, the data analyzed here were still quite limited. It would be interesting and important to further validate the proposed approaches with more data in different clinical sets.

Brain activities involve spatial-temporal coordinated dynamics of numerous neurons in different regions of the brain, i.e., involve numerous functional brain networks. To better characterize the synergistic effects among the brain networks, it is important to construct brain networks based on multi-channel EEG signals. Closely related to this network issue is to infer the localization of each type of epileptiform discharges, which is of great clinical importance. These issues have not been pursued in this work, which is obviously a serious limitation of the current study. In the near future, we will examine these issues systematically, especially from the viewpoint of synthesizing network analysis with nonlinear analysis based on complexity science.

## Data Availability Statement

The raw data supporting the conclusions of this article will be made available by the authors, without undue reservation.

## Ethics Statement

The scalp EEG data analyzed here were clinically obtained at the First Affiliated Hospital to Guangxi Medical University. The studies involving human participants were reviewed and approved by the ethics committee of the First Affiliated Hospital to Guangxi Medical University. The participants provided their written informed consent to participate in this study.

## Author Contributions

QL performed most of the experimental work. QH and YW provided the data needed for this experiment and engaged in many discussions, together with BX. JG conceived the study, provided overall supervision for the study, directed all phases of the study, and including writing of the manuscript. All authors read and approved the final manuscript.

## Conflict of Interest

The authors declare that the research was conducted in the absence of any commercial or financial relationships that could be construed as a potential conflict of interest.
